# Efficacy of Timothy grass allergy immunotherapy tablet (AIT) treatment in Canadian children and adults with grass pollen-induced allergic rhinoconjunctivitis (ARC)

**DOI:** 10.1186/1710-1492-7-S2-A17

**Published:** 2011-11-14

**Authors:** Jacques Hebert, Hendrik Nolte, Jennifer Maloney, Michael Blaiss, Harold S Nelson

**Affiliations:** 1 Centre de Recherche Appliquee en Allergie de Quebec, Quebec, Canada; 2 Merck & Co., Kenilworth NJ, USA; 3 Departments of Pediatrics and Medicine, University of Tennessee Health Science Center, Memphis TN, USA; 4 Department of Medicine, Division of Allergy and Immunology, National Jewish Health, Denver CO, USA

## Background

The effect of Timothy grass AIT treatment on a Canadian subpopulation was assessed post-hoc using data from 2 randomized, double-blind, trials designed to evaluate grass AIT in North American subjects.

## Methods

Subjects 5 years and older with ARC with/without asthma received once-daily 2800 BAU sublingual grass AIT (oral lyophilisate, *Phleum pratense*, 75,000 SQ-T, ~15μg Phl p5) or placebo starting approximately 16 weeks before and continuing throughout the 2009 grass pollen season (GPS). Subjects used daily e-diaries to record ARC symptoms and use of symptomatic medications from randomization through study end (approximately 24 weeks). The primary efficacy endpoint comprised the average total combined daily symptom and medication score (TCS) during the entire GPS. Secondary endpoints included average daily symptom score (DSS) and average daily medication score (DMS). Safety was assessed by monitoring adverse events (AEs).

## Results

The Canadian subpopulation included 103 subjects (46 pediatric [5-17y]; 57 adult [18-65y]). AIT-treated subjects showed reductions, vs placebo, of 38% (*P*=.016) for entire and 45% (*P*=.005) for peak seasons in TCS; of 38% (*P*=.013) for entire and 40% (*P*=.008) for peak seasons in DSS; and of 39% for entire (*P*=.238) and 60% (*P*=.005) for peak seasons in DMS. Among the overall population, most treatment-related AEs were mild or moderate. No serious or life-threatening treatment-related AEs occurred; no new safety concerns emerged.

## Conclusions

Timothy grass AIT, a novel therapeutic modality, significantly improved ARC caused by Timothy grass pollen and related grasses in Canadian adults and children 5 years and older.

